# JQ1 Treatment and miR-21 Silencing Activate Apoptosis of CD44+ Oral Cancer Cells

**DOI:** 10.3390/ijms26031241

**Published:** 2025-01-31

**Authors:** Milica Jaksic Karisik, Milos Lazarevic, Dijana Mitic, Olivera Mitrovic Ajtic, Giuseppe Damante, Jelena Milasin

**Affiliations:** 1 Department of Human Genetics, School of Dental Medicine, University of Belgrade, 11000 Belgrade, Serbia; milica.jaksic@stomf.bg.ac.rs (M.J.K.); milos.lazarevic@stomf.bg.ac.rs (M.L.); dijana.trisic@stomf.bg.ac.rs (D.M.); 2 Department of Molecular Oncology, Institute for Medical Research, National Institute of the Republic of Serbia, University of Belgrade, 11000 Belgrade, Serbia; oliveram@imi.bg.ac.rs; 3 Department of Medical Area, University of Udine, 33100 Udine, Italy; giuseppe.damante@uniud.it

**Keywords:** oral cancer, cancer stem cell, miR-21, JQ1, epigenetic modulation

## Abstract

Oral cancer ranks in the top 10 most prevalent malignancies worldwide. It is an aggressive tumor with frequent relapses and metastases and relatively modest survival rates that do not improve in spite of constantly evolving treatment modalities. Cancer stem cells are a subpopulation of tumor cells considered to be responsible not only for tumor initiation but also its aggressive behavior. Many efforts are directed at targeting those cells specifically. A class of small molecules, inhibitors of BET proteins (iBET), is emerging as a novel anticancer tool. Modulating the expression of microRNAs could also be a valid approach in cancer therapy. We aimed to study the effect of the iBET JQ1 combined with miR-21 silencing on oral cancer stem cells (CD44+ cells). CD44+ cells were sorted by flow cytometry and treated with JQ1 alone or in combination with miRNA-21 silencing. Following treatment, MTT, spheroid formation, invasion, and annexin V assays were performed, along with cell cycle and gene expression analyses. JQ1 in conjunction with miR-21 silencing showed considerable cytotoxicity led to a significant downregulation of cyclin D1, consistent with G1 cell cycle arrest, a significant caspase 3 upregulation in accordance with activation of apoptosis. The combined treatment approach also reduced CD44+ cell invasion capacity. Modulating chromatin structure with iBETs and silencing miRNA could be suitable epigenetic adjuncts to oral cancer treatment.

## 1. Introduction

The recent global statistics indicate that there are more than 350,000 newly reported cases of oral cancer and almost 180,000 deaths per year [1,2]. Oral squamous cell carcinoma (OSCC) accounts for 90% of all head and neck cancer cases, and it is expected that this incidence will increase in the next 15 years [3,4]. It is characterized by a high cellular heterogeneity and its hierarchical organization, with a specific group of cells known as cancer stem cells (CSCs) playing a major role [5]. Cancer stem cells are a small subpopulation of tumor cells that possess self-renewal ability, the capacity to initiate tumor formation, and contribute to the main tumor’s characteristics, including the propensity to recurrence and metastasis, as well as to drug and radiation resistance. Oral cancer CSCs have mostly been isolated using the CD44 marker [6]. CD44, a cell membrane glycoprotein involved in cell–cell interactions, cell adhesion, and migration, is essential for CSC function [7]. Experimentally, reducing CD44 expression causes CSCs to lose their “stemness” traits [8].

Epigenetics has been recognized as essential for the dynamic control of gene expression, encompassing both activation and repression mechanisms. It has been established that epigenetics plays a crucial role in various diseases, including cancer. Epigenetic events comprise DNA methylation, histone modification, and chromatin remodeling and noncoding RNAs [9]. The components involved in different histone modification patterns can be classified into three roles: Writers, Readers, and Erasers [9]. One noteworthy set of Readers, which influence chromatin structure and function either by recruiting other proteins or by blocking Writers and Erasers from accessing specific modifications, are bromodomain and extra-terminal (BET) proteins [10]. They play a crucial role in regulating gene transcription through interactions between bromodomains and acetylated histones. BET blockade leads to selective repression of the transcription; hence, inhibitors of BET (iBETs) are emerging as a novel class of antineoplastic agents [11]. Bromodomain-containing protein 4 (BRD4) is an important member of the BET family with pleiotropic functions (chromatin remodeler via its histone acetyltransferase activity, scaffold for transcription factors, regulator of transcription via its kinase activity, etc.) and thus with the capacity to promote tumor progression by regulating multiple genes. Prior studies have documented that BRD4 inhibitors have substantial anticancer properties across different cancer types, including OSCC [12]. JQ1 belongs to a group of small molecule inhibitors, which competitively binds to BRD4, leading to cell proliferation inhibition and apoptosis promotion [13].

MicroRNAs (miR) are a group of short noncoding RNAs, which possess the ability to control gene expression. They may either inhibit translation or induce transcript degradation [14]. MiRs act as epigenetic regulators, modulating the expression of target messenger RNAs (mRNAs) without modifying the gene sequences. At the same time, miRNAs can also be affected by epigenetic alterations [15]. MicroRNA-21 (miR-21) has been shown to enhance resistance to chemotherapy in numerous types of cancer and has been associated with a poor prognosis [16]. It has been suggested that targeting miR-21 in head and neck squamous cell carcinoma could restore drug sensitivity to chemotherapeutics, for instance, to cisplatin [17].

Over the past decade, numerous studies have highlighted the potential of combining epigenetic agents with traditional chemotherapeutic treatments, positioning epigenetic therapy as a promising direction [18,19]. This strategy has the potential to enhance the sensitivity of cancer cells, including CSCs that have developed resistance to treatment [20,21]. Recent studies have found that JQ1 can affect the expression of miR21 in tumor cells [21], but no studies have yet explored the effect of miR-21 silencing on the treatment of oral CSCs in conjunction with JQ1. Hence, this study aimed to determine whether JQ1 treatment and miR-21 silencing could act synergistically and what was their impact on cell survival, apoptosis, invasion, and the cell cycle.

## 2. Results

### 2.1. CD44+ Cell Sorting

**Figure 1:**
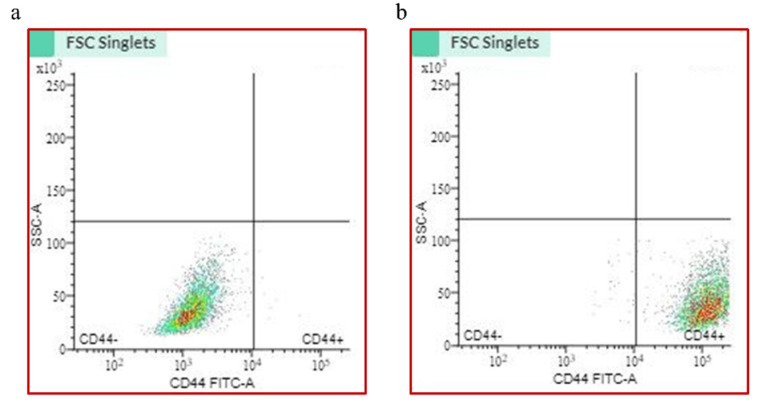
Cell sorting via flow cytometry. (**a**) Dot plot of heterogeneous unsorted cell population; (**b**) dot plot of CD44+ sorted cells.

CD44+ cells were separated from the heterogenous cancer cell cultures using Fluorescence-Activated Cell Sorting (FACS). In the heterogenous primary cancer cell cultures generated from oral cancer patients, only a small subpopulation (below 1%) of cells were CD44+. Following sorting, as much as 99.3% were CD44+ cells (Figure 1).

### 2.2. Expression on miR-21 in CSCs

**Figure 2:**
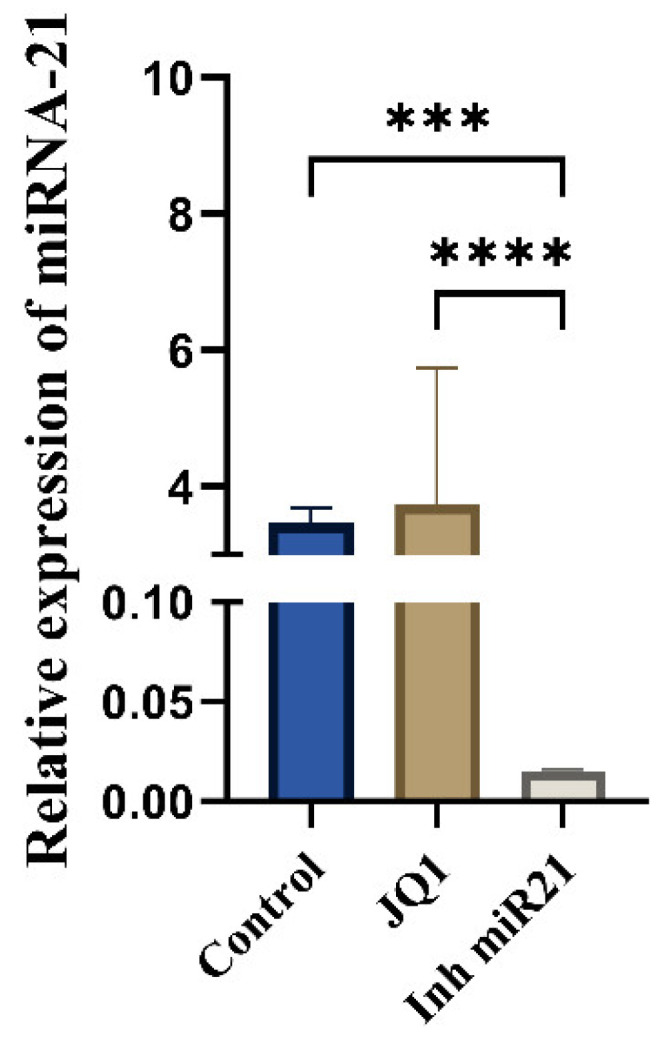
JQ1 does not affect miR-21 expression in cancer stem cells. Relative gene expression of miR-21 in control CD44+ cells and after the treatment with JQ1 and miR-21 inhibition. *** *p* < 0.001; **** *p* < 0.0001.

We analyzed miR-21 expression in the control group of CSCs (CD44+), in JQ1-treated CD44+ cells, and in CD44+ cells transfected with miR-21 inhibitor. A statistically significant change in miR-21 expression was observed only in the group with transfected miR-21 inhibitor (Figure 2). This suggests that JQ1 does not affect miR-21 expression in oral CSCs.

### 2.3. Effect of JQ1 Alone and JQ1 + miR-21 Inhibition on CD44+ Viability

**Figure 3:**
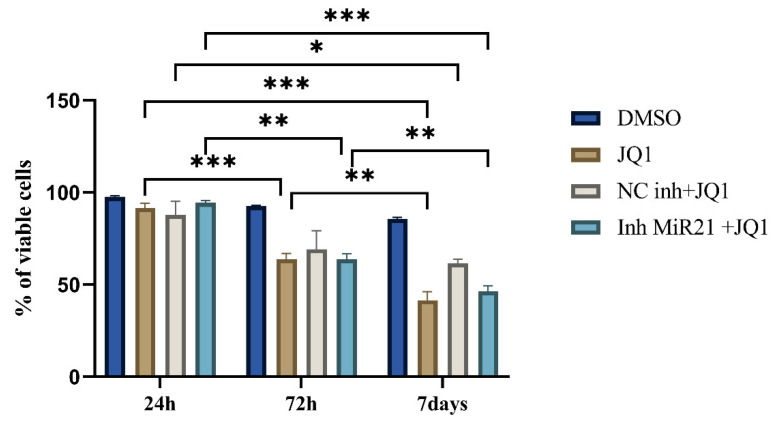
MTT viability assay. Cell proliferation after treatment with DMSO (Control), 10 mM JQ1, and 10 mM of JQ1 after miR-21 inhibition, * *p* < 0.05, ** *p* < 0.01, *** *p* < 0.001.

The cells were treated with 10 mM JQ1 for 1, 3, and 7 days. As shown by MTT, cell proliferation was decreased after 3 and 7 days compared to the control group (cells treated with DMSO). The percentage of viable cells was significantly lower after 7 days (only 45%) of JQ1 treatment compared to 24 h (95%) and 3 days (60%) (*p* < 0.0001) (Figure 3). Based on these results, further experiments were conducted using the 7-day treatment.

In order to evaluate whether the transfection itself could potentially influence the treatment efficiency, we included a control group of cells transfected with the “microRNA inhibitor Negative Control” (NC inh) and then treated with JQ1. There was no statistically significant difference between the three treatment modalities (JQ1 alone, JQ1 + NC inh, and JQ1 combined with miR-21 inhibition). 

### 2.4. Effects of JQ1 Alone and JQ1 + miR-21 Inhibition on CD44+ Cell Apoptosis

**Figure 4:**
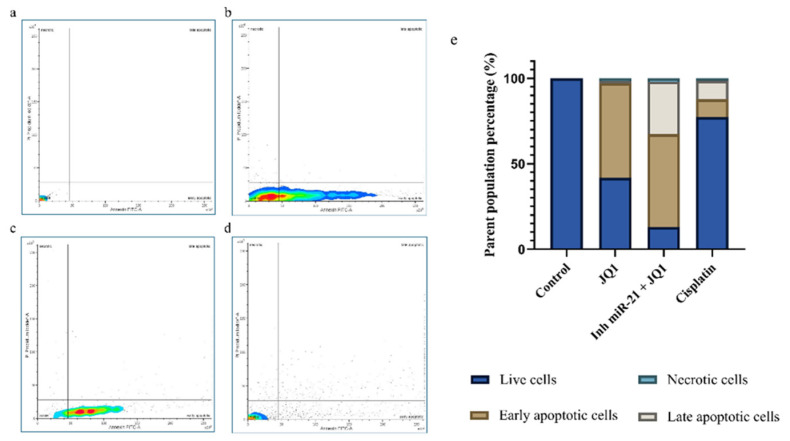
JQ1 and miR-21 silencing effect on apoptosis in CD44+ cells. (**a**) Dot plot of control cells; (**b**) after JQ1 treatment, the highest percentage of cells was found in early apoptosis; (**c**) combined JQ1 treatment and miR-21 silencing showed a higher percentage of apoptotic cells; (**d**) dot plot of cisplatin treated cells; (**e**) results of annexin V assay presented in percentages.

After 7 days of JQ1 treatment, the majority of CD44+ cells (55.2%) were in early apoptosis. Cells subjected to the combined treatment exhibited a similar percentage of early apoptotic cells (54.2%) but showed an increase in late apoptotic cells to 30.6%. Combining the two epigenetic treatments appeared to be much more efficient than JQ1 alone or cisplatin. The percentage of viable cells following JQ1+ miRNA-21 silencing was reduced to 13.1% compared to single JQ1 treatment where it was 44.9% (Figure 4).

### 2.5. JQ1 and miR-21 Inhibition Effect on Cell Cycle of CD44+ Cells

**Figure 5:**
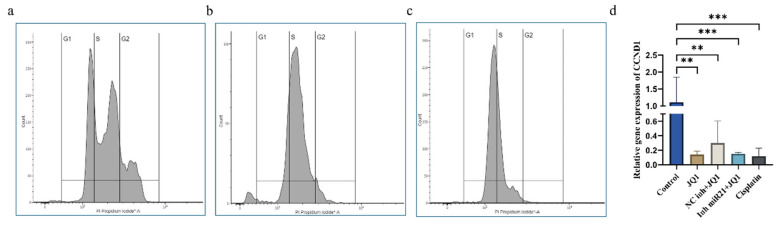
CD44+ cell cycle. (**a**) Cell cycle distribution in CSCs without treatment, (**b**) after treatment with JQ1, and (**c**) in cells with miR-21 inhibition+ JQ1. (**d**) The combined effect of the two epigenetic modulations highly contributes to the reduction in *CCND1* expression, ** *p* < 0.01, *** *p* < 0.001.

To further explore the mechanisms by which JQ1 inhibits CSC proliferation, we evaluated its effects on the cell cycle. JQ1 treatment increased the percentage of cells in G1 (51.8%) and decreased in S phase (44.4%) compared to the control (G1: 37.5%, S: 48.5%). However, JQ1 treatment in conjunction with miR-21 silencing achieved a significant effect by arresting almost 90% (89.4%) of cells in the G1 phase and reducing the number of cells in the S phase to only 10.2%. We used RT-qPCR to examine the expression of *CCND1* gene (encoding cyclin D1), a crucial cell cycle regulator of the G1/S transition, and found that JQ1 alone and concomitantly with miR-21 inhibition had a highly significant effect on *CCND1* downregulation, which is consistent with G1 cell cycle arrest (Figure 5d). To check whether the process of transfection affects treatment effectiveness or not, we included a group of cells transfected with the NC inhibitor and treated with JQ1. There was no statistically significant difference in *CCND1* expression between JQ1-treated cells and cells that were NCinh-transfected prior to JQ1 treatment (Figure 5).

### 2.6. JQ1 Associated with miR-21 Inhibition Effect on Cancer Stem Cell Invasion

**Figure 6:**
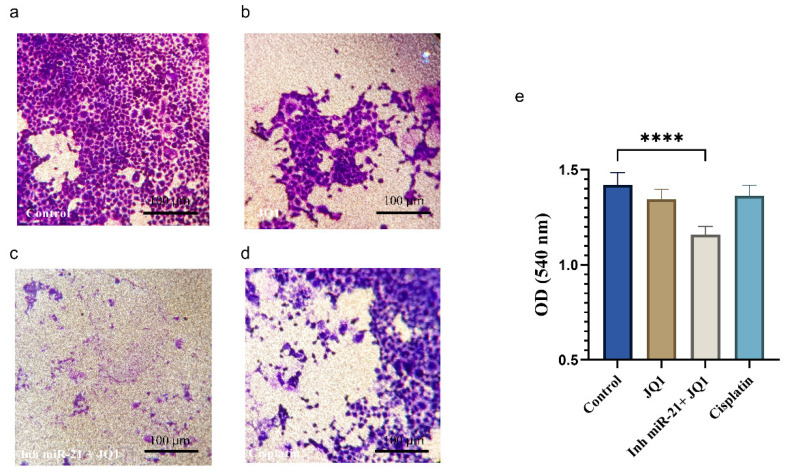
Transwell cell invasion assay. (**a**) Control CD44+; (**b**) JQ1 alone treatment; (**c**) JQ1 + miR-21 inhibition; (**d**) cisplatin; (**e**) quantification of invasion assay (scale bar 100 µm, magnification 10×), **** *p* < 0.0001.

Following the combined treatment of CD44+ cells, their ability to invade was considerably reduced. JQ1 alone did not achieve an important reduction but only in combination with miR-21 silencing, and it proved to be much more efficient than the standard cisplatin treatment (Figure 6).

### 2.7. JQ1 and JQ1+ miR-21 Effect on Apoptosis Inhibition

**Figure 7:**
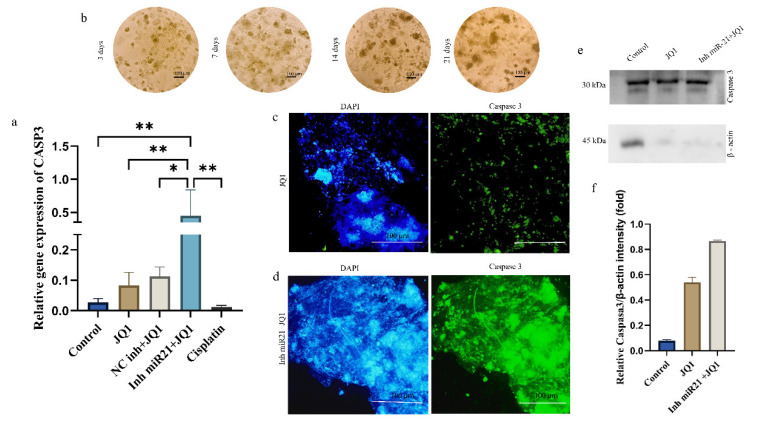
(**a**) Relative gene expression of caspase 3 in CSCs after the treatment with JQ1, JQ1 + NC inh, and JQ1 + miR-21 inhibitor and cisplatin; (**b**) the images of CD44+ cells spheroid formation (at 10× magnification); (**c**) fluorescent microscopy of spheroids stained with DAPI (left) and caspase 3 FITC antibody (right) after treatment with JQ1; (**d**) spheroids stained with DAPI (left) and caspase 3 FITC antibody (right) after treatment with JQ1 + miR-21 silencing; (**e**) Western blot analysis of caspase 3 in the control, JQ1-treated cells, and JQ1 + miR-21 silenced cells; (**f**) quantification of WB results, * *p* < 0.05, ** *p* < 0.01.

Real-time PCR analysis also showed a statistically highly significant upregulation of *CASP3* gene (encoding caspase 3) after JQ1 treatment of miR-21-inhibited cells (15.5-fold compared to control) (Figure 7a). To investigate the effects of JQ1 and miR-21 silencing on apoptosis gene expression (caspase 3), we performed immunofluorescence, Western blot, and RT qPCR analyses. Figure 7b shows images of spheroid cell formations over a period of 21 days. After this period, the spheroids underwent miR-21 silencing and JQ1 treatment. The results of fluorescent microscopy indicate that the spheroids with the combined treatment express much higher levels of caspase 3 (Figure 7c, right) than the cells treated with JQ1 alone (Figure 7d right). Again, in order to exclude the possibility that the process of transfection could impact treatment effectiveness, we included a group of cells transfected with the NC inhibitor and treated with JQ1. There was no statistically significant difference of *CASP3* expression between JQ1-treated cells and cells that were transfected prior to JQ1 treatment. In other words, the transfection itself does not seem to affect the behavior of the antineoplastic agent. The increased expression of caspase 3 (Figure 7e,f) was also confirmed at the protein level.

## 3. Discussion

Multiple epigenetic mechanisms are interconnected and work together to regulate the expression of specific genes. The identification of epigenetic alterations caused by miRNA has brought about a significant transformation in cancer research [22]. Mounting evidence indicates that CD44 acts as a marker for cancer stem cells and has a significant function in controlling CSC characteristics, including self-renewal, tumor initiation, metastasis, and resistance to chemotherapy and radiotherapy [8]. In our previous investigations, we have successfully isolated and characterized CD44+ cells in which the expression of embryonic/cancer stem cell markers *OCT4*, *SOX2*, and *NANOG* was significantly higher than in the CD44− cell populations [23]. Following those results, we aimed to specifically study the CD44+ cells and epigenetically target them.

Histones’ modifications and noncoding RNAs have been shown to have an important role in cancerogenesis, prompting us to explore the potential of targeting two levels of epigenetic regulation to enhance the efficacy of CSC eradication.

Epigenetic readers have the capacity to directly or indirectly control the expression of genes. One example is the bromodomain and extra-terminal (BET) family of proteins (BRD2, BRD3, and BRD4), which have the ability to regulate gene expression by identifying acetylated histones. Blocking the activity of BRD4 has recently been recognized as a potentially effective approach to treat cancer. BET inhibitors such as JQ1 have shown the ability to decrease cell proliferation in oral squamous cell carcinoma [12]. However, JQ1 alone does not produce significant outcomes as a stand-alone treatment [24,25]. In our recently published study, we have examined the various roles of miR-21 in CSCs and demonstrated that the inhibition of miR-21 leads to decreased expression of stemness markers (*OCT4*, *SOX2*, and *NANOG*) and makes the cells more susceptible to apoptosis. The latter effect was attributed to the reduced expression of the antiapoptotic gene *BCL-2* [26]. As miRNA silencing has previously been shown to exert tumor-suppressing effects, we decided to inhibit miRNA-21, a microRNA highly expressed in oral cancer, and aimed to look for a potential synergistic action of two epigenetic alterations (microRNA silencing plus histone modification).

The epigenetic treatments led to an increased cell cycle arrest in G1 phase and a decreased percentage of cells in the S phase. The G1 phase was prolonged in JQ1-treated cells but even more in cells subjected to JQ1+miR-21 silencing. This result was consistent with a reduction in the expression of cyclin D1 protein. Previous research has demonstrated that cyclin D1 is a crucial regulator of cell cycle progression in ovarian cancer cells and that the degradation of cyclin D1 was enough to trigger G1 cell cycle arrest [27].

Studies have established that miRNA-21 is excessively produced in many types of cancer, including oral cancer, and linked to their capacity of invasion [13,27]. Hence, miRNA inhibition is a sound approach in terms of invasion lessening. In the present study, the combination of JQ1 treatment and miRNA-21 silencing has led to a significant reduction in CD44+ cell invasion ability.

Our findings also showed that JQ1 alone has the ability to trigger apoptosis of CD44+ cells but, when miRNA-21 was suppressed prior to JQ1 treatment, the percentage of cell death significantly increased (up to 87%). This was accompanied with upregulation of caspase 3, a key mediator of apoptosis and responsible for the cleavage of many proteins. This finding was in agreement with the study of Murad et al. [28]. Our results demonstrated that the combined treatment, more than JQ1 alone, upregulated *CASP3* gene expression in CD44+ cell cultures (a 15-fold increase of mRNA was noted compared to control cells). This increased expression of *CASP3* mRNA was further strengthened by immunofluorescence on CD44+ cell spheroids.

In summary, we have confirmed the antineoplastic effect of JQ1 on the population of oral CSCs (CD44+ cells). The BET inhibitor JQ1, which modulates epigenetic processes at the histone level, combined with miRNA-21 silencing, effectively led to G1-phase cell cycle arrest via decreased expression of cyclin D1. The combined treatment also induced apoptosis as a consequence of caspase 3 activation. It additionally contributed to the reduction in cancer stem cell invasion capacity. Epigenetic modulations hold great potential in more effective cancer treatment and CSC eradication but further studies are still needed.

## 4. Materials and Methods

### 4.1. Cell Cultures

In this study, six cell cultures were utilized, including the Human OSCC cell line SCC-25, obtained from the American Type Culture Collection (ATCC CRL-1628™), and primary cell cultures generated from tumor tissue samples of five OSCC patients (characteristics of patients are given in Table S1). This study was approved by the Ethical Committee of School of Dental Medicine, University of Belgrade (No. 36/6), and was conducted in accordance with the Declaration of Helsinki. Cells were cultured in T25 cell culture flasks in complete medium (Dulbecco’s Modified Eagle Medium (DMEM) supplemented with 10% of Fetal Bovine Serum (FBS), 100 U/mL penicillin–streptomycin solution, and 400 ng/mL hydrocortisone) under standard conditions in humidified atmosphere with 5% CO_2_ at 37 °C. Complete medium was changed every 2–3 days and, after the cells reached 80% of confluence, the heterogeneous cancer cell population was magnetically sorted. All chemicals were purchased from Invitrogen (Thermo Fisher Scientific).

### 4.2. Cell Sorting and Flow Cytometry

CD44+ cell separation was performed using flow cytometry (BD FACS Melody^TM^) according to the manufacturer’s protocol. Total populations of adherent cells were enzymatically detached and counted. The cell suspension (10^6^) was incubated with 100 µL of CD44 antibody (ref. no. 130-133-985, Miltenyi Biotec, Auburn, CA, USA) at 4 °C for 30 min; then, cells were washed three times with PBS and passed through the FACS system. Experiments were performed on second passage cells. Cells were sorted and their stemness verification via flow cytometry was always conducted prior to the seeding, i.e., the purity of the CD44+ cell culture was ensured before each experiment. Following the separation, characterization of CD44+ cells was conducted, as described in the previous study [23].

### 4.3. MTT Assay

Based on our previous results, we decided to apply JQ1 at a concentration of 10 µM in the present study [12]. Cells were seeded onto 96-well plates (1 × 10^4^ cells/well) and cultured in complete growth medium with 10 µM JQ1 for 24 h, 3, and 7 days. Dimethyl sulfoxide (DMSO) was added in the control (untreated) group. Following the different incubation periods, MTT (3-(4,5-dimethylthiazolyl-2)-2,5 diphenyltetrazolium bromide) was added to each well. After 4 h of incubation, supernatant was discarded and the precipitates were dissolved in 100 µL dimethyl sulfoxide (DMSO) (Sigma-Aldrich) by shaking at 37 °C for 15 min. Optical density (OD) was measured at 540 nm using a Microplate Reader (RT- 2100c, Rayto, Shenzhen, China). The percentage of viable cells was calculated using the following formula:

% of viable cells = OD (sample)/OD (control) × 100(1)

### 4.4. Transfection of miRNA-21 Inhibitor

CD44+ cells were seeded onto 6-well plates (1 × 10^6^ cells per well) and grown to 80% confluence. Cells were transfected with miRNA inhibitors (MISSION Synthetic microRNA inhibitor, Human hsa-miR-21-5p) along with the Negative Control-NC (MISSION Synthetic microRNA inhibitor Negative Control 1) using Lipofectamine RNAiMIX (Invitrogen, Thermo Fisher Scientific, Waltham, MA, USA) in OptiMEM (Invitrogen, Waltham, MA, USA), according to the manufacturer’s instruction. Cells in which only Lipofectamine™ RNAiMAX was added were used as the control group. After 72 h, inhibition of miR-21 and the negative control was confirmed by RT-qPCR using TaqMan assay. Cells from each of the 3 groups (control, NC, and miR-21 inhibitor) were collected to be used for further analysis.

### 4.5. Apoptosis Assay (Annexin V)

CD44+ cells were seeded into 24-well plates (1 × 10^5^ cells per well). CD44+ and CD44+ cells transfected with miR-21 inhibitor were treated with JQ1 at a concentration of 10 μM. The positive control was CD44+ cells treated with cisplatin, and the negative control was untreated cells. After 7 days, staining for apoptosis detection was performed with an Annexin V–FITC Apoptosis Detection Kit (Invitrogen, Thermo Fisher Scientific) according to the manufacturer’s instructions. The Annexin V-FITC staining was analyzed using the flow cytometer BD FACSMelodyTM, and the results were displayed in a two-dimensional dot plot of propidium iodide (PI) versus Annexin V-FITC. PI was used to detect necrotic and late apoptotic cells. The plots were divided into four regions corresponding to (a) viable cells, negative for both probes (PI/FITC −/−; Q3); (b) apoptotic cells, PI-negative and Annexin-positive (PI/FITC −/+; Q1); (c) late apoptotic cells, PI- and Annexin-positive (PI/FITC +/+; Q2); and (d) necrotic cells, PI-positive and Annexin-negative (PI/FITC +/−; Q4).

### 4.6. Transwell Cell Invasion Assay

In the transwell invasion assay, first, the upper chamber was prepared by adding 30 µL of Corning^®^ Matrigel^®^ and incubated at 37 °C for 30 min before seeding 10^5^ cells per well. Cells were resuspended in 500 µL of a culture medium (without 10% FBS) with or without 10 μM of JQ1. Into the lower chamber of the transwell plate, the complete culture medium with 10% of FBS (used as chemoattractant) was added. The control chamber contained only the complete growth medium. The dish was subsequently incubated for 24 h to facilitate cellular invasion. Following the incubation period, non-migrated cells on the upper side of the membrane were removed using a cotton swab and the cells located at the bottom of the top chamber were treated with a 4% paraformaldehyde solution for a duration of 30 min. Afterwards, they were treated with a 0.2% Crystal Violet solution for an additional 30 min. The cells were imaged using microscopy, and the resulting images were subsequently analyzed by ImageJ software 1.48 version (NIH, Bethesda, MD, USA) [25]. For colorimetric quantification of invasion assay, the wells were filled with 750 μL of 10% acetic acid and incubated for 30 s while carefully shaking the 24-well plate. The cells on the membrane were lysed with acetic acid, and Crystal Violet was released from the cells. The insert was then removed from the 24-well plate and 10% acetic acid was transferred to a 96-well plate and read using a microplate reader at the optical density of the at 540 nm [29].

### 4.7. Cell Cycle

CD44+ cells were seeded in 6-well plates to ensure a minimum of 10^6^ cells per test sample. After 7 days of treatment with JQ1, cells were centrifuged at 1400 rpm for 6 min and washed with PBS for 5 min. After another centrifugation cycle, the cells were suspended in 300 µL of PBS. Gradually, 700 µL of 96% ice-cold ethanol was added dropwise, and incubated for a period of 2 h at 4 °C. Following centrifugation at 1700 rpm for 6 min, ethanol was removed, and cells resuspended in PBS. After another round of centrifugation, the cells were resuspended in 500 µL of PBS, and 7 µL of RNase A (100 µg/mL) was added, followed by a 15-min incubation at 37 °C. Just before analysis on the flow cytometer, PI was added at a final concentration of 50 µg/mL. Finally, the percentages of cells in different phases of the cell cycle were determined using the flow cytometer BD FACSMelody^TM^ and BD FACSChorus^TM^ software (version 3.0).

### 4.8. Isolation of RNA and Reverse-Transcription Polymerase Chain Reaction

Total RNA was extracted from the cells using a TRIzol Reagent (Invitrogen, Thermo Fisher Scientific), according to the manufacturer’s recommendations. The concentration of RNA was measured using a microvolume spectrophotometer (BioSpec–nano Microvolume UV–Vis Spectrophotometer (Bartlesville, OK, USA); Shimadzu Scientific Instruments, Columbia, MD, USA). An oligo d(T) primer and RevertAid First Strand cDNA Synthesis Kit (Thermo Fisher Scientific, Waltham, MA, USA) were used to synthesize cDNA from 2 µg of total RNA [30]. For assessing the miR–21 expression level in cells, RNA was isolated from cells using the same protocol.

### 4.9. Real-Time Quantitative Polymerase Chain Reaction

Gene expression analysis by real-time quantitative polymerase chain reaction (qPCR) was performed using 2 µL cDNA, 0.75 µM forward and reverse primers, 7.5 µL of SensiFAST SYBR Hi–ROX Kit (Bioline, London, UK), and 4 µL of water. Relative expressions of *CASP3* and *CCND1* were analyzed. The reference gene was glyceraldehyde-3-phosphate dehydrogenase—*GAPDH*. Gene expression values were calculated using the 2^−∆Ct^ method. All the primer sequences used in this study are given in Table S2.

TaqMan microRNA assay was conducted to assess the level of miR-21 in CD44+ cell cultures before and after treatment with JQ1. Reverse transcription was performed using 15 µL reactions that consisted of 10× a Reverse Transcription Buffer, an RNase inhibitor, 100 mM deoxyribonucleotide triphosphate (dNTP), and a Multi Scribe Reverse Transcriptase and containing 3 μL of a 5× concentrate miR-21 specific primer. Settings for thermal cycler were 16 °C for 30 min, 42 °C for 30 min, and 85 °C for 5 min, followed by cooling to 4 °C. Quantitative polymerase chain reaction (qPCR) was conducted in 20 μL reactions using a TaqMan 20× concentrate of miR-21 assays (Applied Biosystems, Thermo Fisher Scientific, Waltham, MA, USA), Universal PCR Master Mix, and 2 µL of product from the reverse transcription reaction. Thermal cycler settings were 50 °C for two minutes, 95 °C for 10 min, then 40 cycles of 95 °C for 15 s, and 60 °C for 60 s. RNU44 was used as the internal control for miRNA. The fold change was calculated based on the threshold cycle (Ct) value using the formula: Relative Quantity (RQ) = 2^−ΔΔCT^.

### 4.10. Western Blot Assay

For the Western blot assay, cells were seeded in 6-well plates and treated with JQ1 for 24 h. Cells were lysed at 4 °C for 20 min in 400 µL of lysis buffer (1% NP-40, 0.5% Triton-X100 in PBS, 1 mM Na_3_VO_4_, 10 mM NaF, 10 mM EDTA, and protease inhibitors). Protein concentrations were measured using a NanoDrop spectrophotometer. Proteins were separated by SDS-PAGE and transferred onto Hybond nitrocellulose membranes (GE Healthcare, Buckinghamshire, UK). Membranes were blocked with 4% non-fat milk and 0.5% Tween 20 in PBS, then incubated with a specific caspase 3 primary antibody, at a dilution of 1:2000 (E-AB-60017, Polyclonal, Rabbit), followed by horseradish peroxidase-conjugated secondary antibodies (Sigma-Aldrich, Rockville, MD, USA). Protein bands were detected using an enhanced chemiluminescence system and quantified using a ChemiDoc Imager (version 3.0.1) and ImageLab software (6.1) (Bio-Rad, Hercules, CA, USA) [31].

### 4.11. Immunofluorescence

Immunofluorescence analysis was performed to detect caspase 3. Briefly, 10^3^ cells were mixed with Matrigel, seeded in 96-well plates, and incubated for 30 min. Cells were cultured in 3D-specialized media (DMEM/F-12, N2, B27, 15 mM HEPES, 2 mM L-glutamine, and penicillin–streptomycin supplemented with 100 ng/mL EGF) for three weeks [32]. After that period, spheroids were treated with JQ1 for 72 h (with prior miR-21 inhibition via transfection in the respective group). After treatment, the medium was carefully removed to avoid aspirating the spheres. The spheres were then fixed with 4% paraformaldehyde for 45 min, gently washed with PBS, transferred into tubes, and permeabilized with 0.2% Triton X-100 for 5 min [33]. Spheroids were incubated with caspase 3 primary antibodies (E-AB-60017, Polyclonal, Rabbit) at a dilution of 1:1500 for 24 h, followed by fluorescently labeled secondary antirabbit antibody (dilution 1:1500) for 1 h and 1 µg/mL DAPI the next day. Samples were visualized and photographed using an epifluorescence microscope (Olympus, Tokyo, Japan) [34].

### 4.12. Statistical Analysis

The data analysis was performed using the statistical software GraphPad Prism version 9.0 (GraphPad Software, Inc., La Jolla, CA, USA). The normality of the distribution was confirmed by a Kolmogorov–Smirnov test. To identify statistical differences between groups, a one-way ANOVA test was applied, followed by Dunnett’s multiple comparison test. The data are shown as the mean ± SD. * *p* < 0.05, ** *p* < 0.01, *** *p* < 0.001, and **** *p* < 0.0001 denote statistical significance, which was established at *p* < 0.05. All experiments were conducted in triplicate and repeated at least two times.

## Data Availability

The original contributions presented in this study are included in the article/Supplementary Materials. Further inquiries can be directed to the corresponding author.

## Supplementary Materials

The following supporting information can be downloaded at: https://www.mdpi.com/article/10.3390/ijms26031241/s1.

## References

[1] Bray F., Ferlay J., Soerjomataram I., Siegel R.L., Torre L.A., Jemal A. (2018). Global Cancer Statistics 2018: GLOBOCAN Estimates of Incidence and Mortality Worldwide for 36 Cancers in 185 Countries. CA Cancer J. Clin..

[2] Bugshan A., Farooq I. (2020). Oral Squamous Cell Carcinoma: Metastasis, Potentially Associated Malignant Disorders, Etiology and Recent Advancements in Diagnosis. F1000Research.

[3] Badwelan M., Muaddi H., Ahmed A., Lee K.T., Tran S.D. (2023). Oral Squamous Cell Carcinoma and Concomitant Primary Tumors, What Do We Know? A Review of the Literature. Curr. Oncol..

[4] Barsouk A., Aluru J.S., Rawla P., Saginala K., Barsouk A. (2023). Epidemiology, Risk Factors, and Prevention of Head and Neck Squamous Cell Carcinoma. Med. Sci..

[5] Islam F., Qiao B., Smith R.A., Gopalan V., Lam A.K.-Y. (2015). Cancer Stem Cell: Fundamental Experimental Pathological Concepts and Updates. Exp. Mol. Pathol..

[6] Rodini C.O., Lopes N.M., Lara V.S., Mackenzie I.C. (2017). Oral Cancer Stem Cells—Properties and Consequences. J. Appl. Oral Sci..

[7] Chen X.-J., Zhang X.-Q., Liu Q., Zhang J., Zhou G. (2018). Nanotechnology: A Promising Method for Oral Cancer Detection and Diagnosis. J. Nanobiotechnol..

[8] Yan Y., Zuo X., Wei D. (2015). Concise Review: Emerging Role of CD44 in Cancer Stem Cells: A Promising Biomarker and Therapeutic Target. Stem Cells Transl. Med..

[9] Xu X., Peng Q., Jiang X., Tan S., Yang Y., Yang W., Han Y., Chen Y., Oyang L., Lin J. (2023). Metabolic Reprogramming and Epigenetic Modifications in Cancer: From the Impacts and Mechanisms to the Treatment Potential. Exp. Mol. Med..

[10] Cheng Y., He C., Wang M., Ma X., Mo F., Yang S., Han J., Wei X. (2019). Targeting Epigenetic Regulators for Cancer Therapy: Mechanisms and Advances in Clinical Trials. Signal Transduct. Target. Ther..

[11] Wang N., Ma T., Yu B. (2023). Targeting Epigenetic Regulators to Overcome Drug Resistance in Cancers. Signal Transduct. Target. Ther..

[12] Baldan F., Allegri L., Lazarevic M., Catia M., Milosevic M., Damante G., Milasin J. (2019). Biological and Molecular Effects of Bromodomain and Extra-Terminal (BET) Inhibitors JQ1, IBET-151, and IBET-762 in OSCC Cells. J. Oral. Pathol. Med..

[13] Jiang J., Yang P., Guo Z., Yang R., Yang H., Yang F., Li L., Xiang B. (2016). Overexpression of microRNA-21 Strengthens Stem Cell-like Characteristics in a Hepatocellular Carcinoma Cell Line. World J. Surg. Oncol..

[14] Zhang B., Pan X., Cobb G.P., Anderson T.A. (2007). microRNAs as Oncogenes and Tumor Suppressors. Dev. Biol..

[15] Yao Q., Chen Y., Zhou X. (2019). The Roles of microRNAs in Epigenetic Regulation. Curr. Opin. Chem. Biol..

[16] Valeri N., Gasparini P., Braconi C., Paone A., Lovat F., Fabbri M., Sumani K.M., Alder H., Amadori D., Patel T. (2010). MicroRNA-21 Induces Resistance to 5-Fluorouracil by down-Regulating Human DNA MutS Homolog 2 (hMSH2). Proc. Natl. Acad. Sci. USA.

[17] Sheng S., Su W., Mao D., Li C., Hu X., Deng W., Yao Y., Ji Y. (2022). MicroRNA-21 Induces Cisplatin Resistance in Head and Neck Squamous Cell Carcinoma. PLoS ONE.

[18] Asgar M.A., Senawong G., Sripa B., Senawong T. (2016). Synergistic Anticancer Effects of Cisplatin and Histone Deacetylase Inhibitors (SAHA and TSA) on Cholangiocarcinoma Cell Lines. Int. J. Oncol..

[19] Yu X., Zhao H., Wang R., Chen Y., Ouyang X., Li W., Sun Y., Peng A. (2024). Cancer Epigenetics: From Laboratory Studies and Clinical Trials to Precision Medicine. Cell Death Discov..

[20] Sharma P.C., Gupta A. (2020). MicroRNAs: Potential Biomarkers for Diagnosis and Prognosis of Different Cancers. Transl. Cancer Res..

[21] Wan Y., Hoyle R.G., Xie N., Wang W., Cai H., Zhang M., Ma Z., Xiong G., Xu X., Huang Z. (2021). A Super-Enhancer Driven by FOSL1 Controls miR-21-5p Expression in Head and Neck Squamous Cell Carcinoma. Front. Oncol..

[22] Muñoz P., Iliou M.S., Esteller M. (2012). Epigenetic Alterations Involved in Cancer Stem Cell Reprogramming. Mol. Oncol..

[23] Jaksic Karisik M., Lazarevic M., Mitic D., Nikolic N., Milosevic Markovic M., Jelovac D., Milasin J. (2023). Osteogenic and Adipogenic Differentiation Potential of Oral Cancer Stem Cells May Offer New Treatment Modalities. Int. J. Mol. Sci..

[24] Filippakopoulos P., Qi J., Picaud S., Shen Y., Smith W.B., Fedorov O., Morse E.M., Keates T., Hickman T.T., Felletar I. (2010). Selective Inhibition of BET Bromodomains. Nature.

[25] Liu H., Guo H., Wu Y., Hu Q., Hu G., He H., Yin Y., Nan X., Lin G., Han J. (2023). RCN1 Deficiency Inhibits Oral Squamous Cell Carcinoma Progression and THP-1 Macrophage M2 Polarization. Sci. Rep..

[26] Jaksic Karisik M., Lazarevic M., Mitic D., Milosevic Markovic M., Riberti N., Jelovac D., Milasin J. (2025). MicroRNA-21 as a Regulator of Cancer Stem Cell Properties in Oral Cancer. Cells.

[27] Masamha C.P., Benbrook D.M. (2009). Cyclin D1 Degradation Is Sufficient to Induce G1 Cell Cycle Arrest despite Constitutive Expression of Cyclin E2 in Ovarian Cancer Cells. Cancer Res..

[28] Murad H., Hawat M., Ekhtiar A., AlJapawe A., Abbas A., Darwish H., Sbenati O., Ghannam A. (2016). Induction of G1-Phase Cell Cycle Arrest and Apoptosis Pathway in MDA-MB-231 Human Breast Cancer Cells by Sulfated Polysaccharide Extracted from Laurencia Papillosa. Cancer Cell Int..

[29] Huang Y., Zhao N. (2020). Generalized Anxiety Disorder, Depressive Symptoms and Sleep Quality during COVID-19 Outbreak in China: A Web-Based Cross-Sectional Survey. Psychiatry Res..

[30] Livak K.J., Schmittgen T.D. (2001). Analysis of Relative Gene Expression Data Using Real-Time Quantitative PCR and the 2(-Delta Delta C(T)) Method. Methods.

[31] Kapor S., Vukotić M., Subotički T., Đikić D., Mitrović Ajtić O., Radojković M., Čokić V.P., Santibanez J.F. (2021). Hydroxyurea Induces Bone Marrow Mesenchymal Stromal Cells Senescence and Modifies Cell Functionality In Vitro. J. Pers. Med..

[32] Múnera J.O., Sundaram N., Rankin S.A., Hill D., Watson C., Mahe M., Vallance J.E., Shroyer N.F., Sinagoga K.L., Zarzoso-Lacoste A. (2017). Differentiation of Human Pluripotent Stem Cells into Colonic Organoids via Transient Activation of BMP Signaling. Cell Stem Cell.

[33] Bergdorf K.N., Phifer C.J., Bechard M.E., Lee M.A., McDonald O.G., Lee E., Weiss V.L. (2021). Immunofluorescent Staining of Cancer Spheroids and Fine-Needle Aspiration-Derived Organoids. STAR Protoc..

[34] Bjelica S., Diklić M., Đikić D., Kovačić M., Subotički T., Mitrović-Ajtić O., Radojković M., Čokić V., Santibanez J.F. (2019). Hydroxyurea-Induced Senescent Peripheral Blood Mesenchymal Stromal Cells Inhibit Bystander Cell Proliferation of JAK2V617F-Positive Human Erythroleukemia Cells. FEBS J..

