# Correction: Jaksic Karisik et al. JQ1 Treatment and miR-21 Silencing Activate Apoptosis of CD44+ Oral Cancer Cells. *Int. J. Mol. Sci.* 2025, *26*, 1241

**DOI:** 10.3390/ijms27020854

**Published:** 2026-01-15

**Authors:** Milica Jaksic Karisik, Milos Lazarevic, Dijana Mitic, Olivera Mitrovic Ajtic, Giuseppe Damante, Jelena Milasin

**Affiliations:** 1Department of Human Genetics, School of Dental Medicine, University of Belgrade, 11000 Belgrade, Serbia; milica.jaksic@stomf.bg.ac.rs (M.J.K.); milos.lazarevic@stomf.bg.ac.rs (M.L.); dijana.trisic@stomf.bg.ac.rs (D.M.); 2Department of Molecular Oncology, Institute for Medical Research, National Institute of the Republic of Serbia, University of Belgrade, 11000 Belgrade, Serbia; oliveram@imi.bg.ac.rs; 3Department of Medical Area, University of Udine, 33100 Udine, Italy; giuseppe.damante@uniud.it

## Text Correction

There was an error in the original publication [1]. In the Results section (Section 2.1), the term “magnetic” was mistakenly used in the sentence “Following magnetic sorting, as much as 99.3% were CD44+ cells.” Flow cytometry and the antibodies used do not operate via the principle of magnetic particles; therefore, the redundant terminology should be removed.

Furthermore, the title of Section 4.2 contained an unnecessary prefix “Magnetic,” which should be removed. Additionally, within the same section, the catalog number of the CD44 antibody was incorrect and needs to be updated to the correct reference number (ref. no. 130-133-985). A correction has been made to Results, Section 2.1, Materials and Methods, and Section 4.2, Paragraph 1.


*2.1. CD44+ Cell Sorting*


CD44+ cells were separated from the heterogenous cancer cell cultures using Fluorescence-Activated Cell Sorting (FACS). In the heterogenous primary cancer cell cultures generated from oral cancer patients, only a small subpopulation (below 1%) of cells were CD44+. Following sorting, as much as 99.3% were CD44+ cells (Figure 1).


*4.2. Cell Sorting and Flow Cytometry*


CD44+ cell separation was performed using flow cytometry (BD FACS Melody^TM^) according to the manufacturer’s protocol. Total populations of adherent cells were enzymatically detached and counted. The cell suspension (10^6^) was incubated with 100 µL of CD44 antibody (ref. no. 130-133-985, Miltenyi Biotec, Auburn, CA, USA) at 4 °C for 30 min; then, cells were washed three times with PBS and passed through the FACS system.

## References

The authors would like to remove reference 19 [2] from the manuscript, as it has been retracted. Its removal does not affect the formulation or conclusions. With this correction, the order of some references has been adjusted accordingly.

The authors state that the scientific conclusions are unaffected. This correction was approved by the Academic Editor. The original publication has also been updated.
