# Supplementary material for: JQ1 Treatment and miR-21 Silencing Activate Apoptosis of CD44+ Oral Cancer Cells
Source: Int J Mol Sci. 2025 Jan 31;26(3):1241. doi: 10.3390/ijms26031241 (PMC11818616; doi:10.3390/ijms26031241)
Supplement: Supplementary file 1 [file ijms-26-01241-s001.zip › ijms-3412948-supplementary.pdf]

Table S1 Primer Sequences used in the study.

GENE	DIRECTION	SEQUENCE
**CCND1**	Rv	5´GGGTGTGCAAGCCAGGTCCA 3´
	Fw	5´ CGGAGGAGAACAAACAGATC 3´
**CASP3**	Rv	5’ CACGCCATGTCATCATCAAC 3’
	Fw	5’ TGTTTGTGTGCTTCTGAGCC 3’
**GAPDH**	Rv	5’ CCC TGT TGC TGT AGC CAA ATT CGT 3’
	Fw	5’ TCA TGA CCA CAG TCC ATG CCA TCA 3’

Table S2. Characteristics of patients which samples were and used for the generation of primary OSCC cell cultures.

**Variables** **Patiente**	**Gender**	**Age** (years)	**Localization**	**TNM status**	**Relapse** Yes/No	**Tobaco/Alcohol consumption** Yes/No	**5-year survival** Yes/No
Patient 1	Female	55	Floor of the mouth	T1-2/N1/M0	No	Yes/Yes	Yes
Patient 2	Male	53	Tongue	T1-2/N0/M0	No	Yes/No	No
Patient 3	Male	58	Maxillary alveolar ridge	T4a/N0/M0	No	Yes/No	No
Patient 4	Male	51	Maxillary alveolar ridge	T4a/N0/M0	No	Yes/No	No
Patient 5	Female	70	Tongue	T3/N0/M0	No	Yes/No	Yes
